# Metabolomics Analysis of the Toxic Effects of the Production of Lycopene and Its Precursors

**DOI:** 10.3389/fmicb.2018.00760

**Published:** 2018-05-03

**Authors:** April M. Miguez, Monica P. McNerney, Mark P. Styczynski

**Affiliations:** School of Chemical & Biomolecular Engineering, Georgia Institute of Technology, Atlanta, GA, United States

**Keywords:** metabolomics, precision metabolic engineering, lycopene, mevalonate, gas chromatography-mass spectrometry, biosensors

## Abstract

Using cells as microbial factories enables highly specific production of chemicals with many advantages over chemical syntheses. A number of exciting new applications of this approach are in the area of precision metabolic engineering, which focuses on improving the specificity of target production. In recent work, we have used precision metabolic engineering to design lycopene-producing *Escherichia coli* for use as a low-cost diagnostic biosensor. To increase precursor availability and thus the rate of lycopene production, we heterologously expressed the mevalonate pathway. We found that simultaneous induction of these pathways increases lycopene production, but induction of the mevalonate pathway before induction of the lycopene pathway decreases both lycopene production and growth rate. Here, we aim to characterize the metabolic changes the cells may be undergoing during expression of either or both of these heterologous pathways. After establishing an improved method for quenching *E. coli* for metabolomics analysis, we used two-dimensional gas chromatography coupled to mass spectrometry (GCxGC-MS) to characterize the metabolomic profile of our lycopene-producing strains in growth conditions characteristic of our biosensor application. We found that the metabolic impacts of producing low, non-toxic levels of lycopene are of much smaller magnitude than the typical metabolic changes inherent to batch growth. We then used metabolomics to study differences in metabolism caused by the time of mevalonate pathway induction and the presence of the lycopene biosynthesis genes. We found that overnight induction of the mevalonate pathway was toxic to cells, but that the cells could recover if the lycopene pathway was not also heterologously expressed. The two pathways appeared to have an antagonistic metabolic effect that was clearly reflected in the cells’ metabolic profiles. The metabolites homocysteine and homoserine exhibited particularly interesting behaviors and may be linked to the growth inhibition seen when the mevalonate pathway is induced overnight, suggesting potential future work that may be useful in engineering increased lycopene biosynthesis.

## Introduction

The use of cells as microbial factories has significant potential in many different contexts. The intricate enzyme machinery available in nature enables highly specific production of molecules ranging from specialty fuels to pharmaceutical precursors. These approaches also have the potential to be more environmentally friendly and sustainable than synthetic chemistry and petrochemical approaches, in aspects ranging from solvent to energy usage. The field of metabolic engineering continues to advance the forefront of what chemicals cells are capable of synthesizing and how much they are capable of making, with ever-increasing titers of ever-more-complex molecules.

Metabolomics, the systems-scale study of the biochemical intermediates of metabolism, can help to inform the development of metabolically engineered strains (Dromms and Styczynski, 2012) through characterization of key endpoints and small molecule regulators of cellular state (Su and Styczynski, 2015; McNerney and Styczynski, 2017b). Using advanced analytical techniques including mass spectrometry, complex mixtures of intracellular small molecules can be quantitatively measured and tracked across samples. Some of these tracked analytes can also be annotated as specific identified metabolites, allowing for biochemical and biological interpretation of the observed metabolic phenotypes.

While metabolomics has proven to be useful in complementing the development of strains designed to maximize biochemical production (Hasunuma et al., 2011; Ito et al., 2014; Gold et al., 2015), it has been largely unexplored in a new area of metabolic engineering that has been growing in prominence: precision metabolic engineering (McNerney et al., 2015). This approach to metabolic engineering focuses less on maximizing total target production and more on increasing the specificity of target production. Examples of precision approaches include dynamic pathway regulation using quorum sensing tools to prevent growth toxicity during product formation (Gupta et al., 2017) and the selective production of only one chemical over another in the presence of specific external stimuli (Watstein et al., 2015). These approaches have the potential to broaden the applications of metabolic engineering beyond traditional industrial-scale fermenters into more portable contexts.

In recent work we have sought to apply precision metabolic engineering approaches to the development of low-cost, minimal-equipment biosensors for application in the developing world (Watstein and Styczynski, 2017). Specifically, we have used *E. coli* as the chassis organism for a whole-cell biosensor to measure zinc status in blood samples, with biosynthesis of differently colored pigments used as the readout for the sensor. In this application, the key challenges to date have included the precise control of production of only one pigment at a time, and the complete repression of any pigment production to enable a dense, colorless initial inoculum that allows a rapid output response (McNerney and Styczynski, 2017a). In this most recent work, we pursued multiple strategies to limit unwanted pigment production and speed up visible pigment production once desired, including precursor supplementation.

Specifically, we engineered strains that produced negligible visible lycopene in the uninduced state and produced unmistakably visible lycopene within approximately 3 h after induction. To attempt to speed up this response time, we heterologously expressed in the same strain the mevalonate pathway, to overproduce the precursors for lycopene biosynthesis. We expected that by providing a mechanism for increased production of lycopene precursors, we could increase total titers and/or decrease the time necessary for unmistakably visible lycopene production. Furthermore, we expected that by inducing the production of these colorless precursors overnight, the cell would be primed for lycopene production upon induction of the heterologous lycopene biosynthesis pathway.

To our surprise, the effect of mevalonate pathway supplementation on cell growth and specific lycopene production varied greatly depending on the time of supplementation. Specifically, if the mevalonate pathway was induced overnight before inoculation and induction of lycopene biosynthesis, specific lycopene production increased and cell growth decreased significantly. In contrast, if the mevalonate pathway was only induced at the same time as the lycopene biosynthesis pathway, there was minimal impact on cell growth and an even greater increase in specific lycopene production. While toxicity associated with lycopene production has certainly been reported before (Farmer and Liao, 2000; Kim and Keasling, 2001; Yoon et al., 2006), in this case our results suggested that it was not necessarily the total levels of lycopene that were causing growth inhibition. To enable further engineering of this pathway to speed up lycopene production, it was of great interest to characterize the metabolic phenotypes underlying this curious cellular behavior.

In this work, we use metabolomics (via two-dimensional gas chromatography coupled to mass spectrometry, GCxGC-MS) to characterize the underlying metabolic states across these counterintuitive observations. We focus on the central carbon metabolites with broad functional and metabolic impacts that are well-measured by GCxGC-MS. After briefly presenting an improved method for quenching *E. coli* for metabolomics analysis, we first characterize the metabolic state of our baseline lycopene-producing strain in the growth conditions characteristic of our biosensor application. We then study the induction of the mevalonate pathway at inoculation and during the overnight culture before induction, with and without the lycopene biosynthesis genes in the same strain. We briefly consider some of the most noteworthy metabolic changes in the affected strains and conditions, suggesting potential hypotheses for the regulation or mechanisms mediating the observed behaviors, and then test and validate one of those hypotheses via a medium supplementation experiment.

## Materials and Methods

### Strains Characterized

*Escherichia coli* K-12 DH10B (New England Biolabs, Ipswich, MA, United States) was used as the host strain for all metabolite production experiments, and all heterologous proteins were expressed from standard expression plasmids. A detailed description of plasmid assembly can be found in McNerney and Styczynski (2017a). In short, the lycopene pathway genes *crtE*, *crtB*, and *crtI* were amplified from Part bba_k274100 of the Registry of Standard Biological Parts and placed under the control of an IPTG-inducible promoter. The mevalonate pathway genes were amplified from the plasmid pJBEI-6409 (Alonso-Gutierrez et al., 2013), which was obtained from Addgene (Cambridge, MA, United States) and placed under control of the arabinose-inducible promoter pBAD to create the plasmid *pBadMEV*. The high lycopene-producing plasmid *pLac32EBI* has the medium-strength RBS B0032 on the *crtE*, *crtB*, and *crtI* genes, and the weak lycopene-producing strain *pLac33EBI* has the weak RBS B0033 on the *crtE*, *crtB*, and *crtI* genes. In all experiments comparing the effect of mevalonate pathway supplementation, the high lycopene-producing plasmid was used. Plasmids *pLacØ* and *pBadØ* were constructed as controls and contain no coding sequence after the promoter.

### Cell Culture

In experiments comparing the metabolic changes based on lycopene production levels, cells were transformed with individual plasmids. In experiments comparing the effect of the mevalonate pathway, cells were co-transformed with two plasmids. Following transformation, cells were plated on LB plates with appropriate antibiotics for selection and grown at 37°C overnight. Freshly transformed colonies were then inoculated in triplicate into LB medium with the appropriate inducers and grown at 37°C and 180 rpm for 18 h. Cultures were then concentrated and inoculated to an OD of 0.2 in fresh medium, the appropriate inducers were added, and the sample was aliquoted into culture tubes corresponding to different time points. Samples were analyzed at the time of inoculation and at hours 1, 2, 4, and 6. At each time point, optical density and lycopene content were measured, and samples were collected for metabolomics analysis. Optical density was quantified by measuring the absorbance at 600 nm in a ThermoFisher Genesys 20 spectrophotometer with a 10 mm path length.

An additional triplicate set of *pLac32EBI + pBadØ* samples as well as two triplicate sets of wild type were grown during the lycopene production comparison experiment to control for variation between experiment days. Lycopene extractions and quenching of these samples were performed with those from the lycopene production comparison experiment, but one set of wild type samples as well as the additional set of *pLac32EBI + pBadØ* samples were stored in a -80°C freezer until extraction of the second experiment’s samples.

LB medium composed of 10 g L^-1^ NaCl, 5 g L^-1^ yeast extract, and 10 g L^-1^ tryptone was used in all experiments. Either 1 mM IPTG or 0.01% (w/v) arabinose was used for induction, and the following antibiotics were used for appropriate selection: tetracycline (15 μg/mL), kanamycin (30 μg/mL), and carbenicillin (100 μg/mL).

### Lycopene Extraction and HPLC Analysis

Lycopene was extracted from cultures and analyzed as described previously (McNerney and Styczynski, 2017a). Briefly, 500 μL of bacterial culture was pelleted and resuspended in 50 μL of ultrapure water. Lycopene was extracted with 1 mL of acetone at 50°C for 20 min. Cellular debris was pelleted, and the supernatant was removed for analysis. Sudan I (TCI America, Portland, OR, United States) was used as an internal standard (Xu et al., 2006) and added to the acetone used for extractions at a concentration of 1 μg/mL.

All HPLC analysis was conducted on a Shimadzu Prominence UFLC using an Agilent C18 4.6 mm × 50 mm column with a 5 μm particle size and a Shimadzu photodiode array detector. A solvent ratio of 50:30:20 acetonitrile:methanol:isopropanol was used as the mobile phase (Lv et al., 2016) and run at a flow rate of 1 mL/min with a 25 μL sample injection volume. Absorption was detected at 471 nm. Retention times and peak intensities were compared to an analytical lycopene standard (Millipore Sigma, St. Louis, MO, United States) spiked into control extractions from DH10B cells, and the internal standard Sudan I was used to account for acetone evaporation during the extraction protocol and for instrument drift.

### Quenching

This method is a modified version of the methods in Spura et al. (2009) and Yasid et al. (2016). A quenching solution composed of 30% ethanol (v/v) and 0.6% NaCl (w/v) in a 15 ml or 50 mL conical was cooled to -15°C. Each tube was prefilled with a certain amount of quenching solution for a 2:1 quench:sample ratio based on estimated OD at sampling time.

Samples were taken at 0, 1, 2, 4, and 6 h for both experiments to capture the metabolic profile over a relevant timeframe for lycopene production. The sample was added to each tube and quickly mixed by inversion. Each tube was kept in a -15°C 70% methanol bath until the sample temperature reached -5 to -8°C. The tubes were then centrifuged for 5 min at 3500 rpm at -10°C and transferred back to a -15°C bath. The supernatant was removed and the tubes were frozen in liquid nitrogen. The samples were stored in a -80°C freezer until extraction.

### Metabolite Extraction

The extraction method is a modified version of the freeze-thaw method in Faijes et al. (2007) and Yasid et al. (2016). The cell pellet was resuspended in 500 μL of -80°C methanol and transferred to a 1.5 mL microcentrifuge tube. The suspension was then frozen in liquid nitrogen, thawed on ice, and centrifuged for 2 min at 10,000 *g* at 4°C. The supernatant was collected and stored in a separate microcentrifuge tube. The pellet was again suspended in an additional 500 μL of -80°C methanol, frozen, thawed, and centrifuged under the same conditions as the previous step. The supernatant was collected, and the pellet was resuspended with 250 μL of cold water, undergoing this freeze-thaw process for a final time. Once the supernatant was collected, the pooled supernatants were more accurately normalized to OD and were transferred to a CentriVap to be centrifugally concentrated at 40°C until completely dry. The dried samples were stored in a -80°C freezer for later processing.

### GC-MS Analysis

Before derivatization, the samples were transferred to a CentriVap to be dried at 40°C for 15 min. Samples were derivatized as previously described (Kind et al., 2009; Vermeersch et al., 2014). 10 μL of 20 mg/mL *O*-methylhydroxylamine hydrochloride (MP Biomedicals, LLC, Santa Ana, CA, United States) in pyridine was added to each dried sample and shaken at 1400 rpm for 90 min at 30°C. 90 μL of *N*-methyl-*N*-(trimethylsilyl) trifluoroacetamide (MSTFA) + 1% trimethylchlorosilane (TMCS) (Thermo Scientific, Lafayette, CO, United States) was then added to the samples and shaken at 1400 rpm for 30 min at 37°C. Samples were centrifuged at 21,100 *g* for 3 min, and 50 μL of the supernatant was added to an autosampler vial. Samples were spiked with 0.25 μL of a retention time standard solution composed of fatty acid methyl esters (FAMES) and an internal standard of nonadecanoic acid methyl ester dissolved in dimethylformamide. In parallel, a quality control (QC) sample was prepared by removing 150 μL of extract from each sample and aliquoting 1.15 mL for experiments comparing the metabolic changes based on lycopene production levels. For experiments comparing the effect of the mevalonate pathway, 75 μL was removed. 1.15 mL and 0.65 mL of QC was aliquoted for each experiment respectively, dried, and derivatized with each batch of 9–10 samples. At the beginning of the GC-MS run, the QCs were injected once and repeated again after every 4–5 sample injections to allow for downstream correction for batch effects. A derivatization blank was prepared and run with every batch of samples.

A LECO Pegasus 4D instrument with an Agilent 7683B autosampler, Agilent 7890A gas chromatograph and time-of-flight mass spectrometer (TOF-MS) was used to analyze the samples. The first column was an HP-5, 28 m long × 0.320 mm ID × 0.25 μm film thickness (Agilent, Santa Clara, CA, United States), and the second was an Rtx-200, 1.75 m long × 0.25 mm ID × 0.25 μm film thickness (Restek, Bellefonte, PA, United States). More detailed gas chromatography, autosampler, and mass spectrometry methods are provided in the Supplementary Material.

### Data Analysis

Sample runs were analyzed in ChromaTOF (LECO, St. Joseph, MI, United States) to determine baseline, peak area, and peak identification as described previously (Dhakshinamoorthy et al., 2015; Vermeersch et al., 2015). Briefly, settings included a baseline offset of 0.5, automatic smoothing, 1st dimension peak width of 36 s, 2nd dimension peak width of 0.10 s, and a match of 700 required to combine peaks with a minimum signal-to-noise (S/N) of 5 for all subpeaks. Peaks were required to have a S/N of 10 and have a minimum similarity score of 800 before assigning a name. Unique mass was used for area and height calculation.

MetPP^1^ was used to align the samples (Wei et al., 2013). Sample files and a derivatization reagent blank file were uploaded from ChromaTOF. Unknowns were retained during the peak alignment process. The derivatization reagent blank file was used to subtract peaks resulting from the sample preparation reagents from the corresponding cells’ sample files. On-the-fly alignment was used with manually selected quality control samples as the peak list for primary alignment. Due to the size of the mevalonate induction time variation experiment, MetPP could not handle on-the-fly alignment using all of the quality control samples. Instead, one quality control sample was used from each batch as well as additional quality controls from the first, last, and fifth batches to perform on-the-fly alignment. Peak alignment was performed using the default criteria.

After alignment, further processing of the data was done based on the procedure previously described (Dunn et al., 2011). Batch effects were removed from the data set using LOESS for both experiments. To remove analytes that were not reproducibly detected, analytes for which more than half of the values were missing in the QC samples or for which the QC samples had a coefficient of variance larger than 0.5 were removed from the data set. Then, missing values were manually corrected using small value correction only if all the values were missing in the biological replicates.

Samples from the mevalonate induction time experiment were processed with Combat (Johnson et al., 2007) to remove batch effects that were evident from principal component analysis of the initial data.

Finally, MetaboAnalyst^2^ was used for statistical and pathway analysis (Xia et al., 2012). For both analyses, remaining missing values were k-nearest neighbors (KNN) corrected. Data was filtered using the interquantile range method and then log-transformed using generalized logarithm transformation (base 2) and autoscaled. Differences were considered significant at false discovery rate-corrected *p* < 0.05. The metabolomics datasets for this study have been deposited to Metabolights with the dataset identifier MTBLS642.

All samples from the experiment comparing the metabolic effects of the amount of lycopene production, except for one triplicate wild type set and the pLac32EBI + pBadØ, were extracted, derivatized, and analyzed together. All samples for the experiment comparing the metabolic effect of the mevalonate pathway as well as those excluded from the previous experiment were extracted, derivatized, and analyzed together.

### Methionine Supplementation

A triplicate set of the strains *pLac32EBI + pBadMEV* and *pLac32EBI + pBadØ* were cultured and induced as described above with the exception of the addition of 2 mM methionine to the medium at the start of the experiment. A triplicate control set of the same strains under the same culture and induction conditions were grown without methionine to test for differences in growth due to methionine addition. Each sample’s optical density was measured at the time of inoculation and at hours 1, 2, 4, and 6, and was quantified as described above.

## Results

### Modified Quenching Protocol

Fast quenching of cellular metabolism for metabolomics analysis is critical for acquisition of samples that accurately represent cellular state in culture rather than artifacts induced by sample processing. Fast filtration, cold methanol, and cold ethanol are the three most common quenching methods for *E. coli*. However, due to the time needed for filtration and quenching of each sample, fast filtration is not appropriate for measuring metabolites with high turnover rates (Smart et al., 2010); it has also been reported to cause leakage in Gram-negative bacteria (Bolten et al., 2007). Numerous studies have shown that the cold methanol method also causes serious leakage from the cell (Bolten et al., 2007; Link et al., 2008; Taymaz-Nikerel et al., 2009; Schädel et al., 2011). We thus selected the cold ethanol method by Spura et al. (2009), which causes significantly less leakage.

Following the original protocol, combining a 37°C sample with a -20°C quenching solution at a 1:1 ratio lowers the sample’s temperature only to about 7–10°C instantaneously before further cooling to -5 to -10°C in a cold bath. 7–10°C is clearly insufficient to halt metabolism instantaneously, and the longer the cells spend in quenching solution, the more leakage is likely to occur (de Jonge et al., 2012). To lower this instantaneous post-quenching temperature and reduce the time the cells need to stay in the solution, Yasid et al. (2016) modified Spura’s method by decreasing the quenching solution’s temperature to -35°C. While this resolves the instantaneous quenching temperature issue (yielding temperatures of 1 to -2°C upon quenching), the quench solution is an icy slurry that melts in a non-uniform fashion and thus requires extra manual supervision that would not be feasible at the scale of sampling needed for this experiment.

To avoid these issues, we modified the quenching solution to allow for a rapid sample temperature decrease to about 0°C while avoiding ice slurries and manual supervision of each quenched sample. We increased the ratio of quenching solution volume to sample volume while maintaining the original overall percentage of ethanol and salt in the final quenched sample (40% ethanol/0.8% sodium chloride quenching solution added at a 1:1 ratio to yield a 20% ethanol/0.4% sodium chloride quenched sample). Our modified quenching solution consists of a 30% ethanol (v/v)/0.6% sodium chloride (w/v) solution added at a 2:1 ratio to sample. Although decreasing the amount of ethanol in the solution increases the freezing point to about -20°C, we were able to keep our quenching solution at -15°C without any ice formation while lowering the sample’s temperature upon initial mixing to about 1 to -3°C (**Table 1**).

**Table 1:** Quenching solution comparisons.

	Quenching solution temperature (°C)	Sample temperature (°C)	Temperature after mixing (°C)	STD (°C)
Spura et al. Method	-20	37	8.4	1.140175
Yasid et al. Method	-35	37	-0.4	1.431782
Modified Method	-15	37	-1.1	1.850676

### Lycopene Levels of Selected Strains Have Minimal Impacts on Growth Characteristics

Overcoming the toxicity effects of heterologous expression of the lycopene biosynthesis pathway from *Pantoea ananatis* is a challenge that has been encountered repeatedly in the field of metabolic engineering. In our previous work, we developed *E. coli* strains that could express different levels of lycopene with minimal effects on cell growth by replacing the native ribosomal binding sites (RBSs) on the *crtEBI* genes with RBSs of varying, but weaker, strength (McNerney and Styczynski, 2017a).

Nonetheless, it has been previously reported that systems-scale metabolic changes due to lycopene production can be detected even before lycopene levels are measurable (Styczynski et al., 2007). While it is obvious that metabolites in the lycopene biosynthesis pathway would have different profiles in strains with different lycopene production potential, we were particularly interested in measuring the extent to which metabolic changes were detectable in more central portions of metabolism with broader impact on cell function and phenotype. To this end, we sought to perform GCxGC-MS metabolomics on two different strains, *pLac32EBI* and *pLac33EBI*, with higher and lower (respectively) levels of lycopene production, and a non-lycopene-producing wild type strain.

**FIGURE 1:**
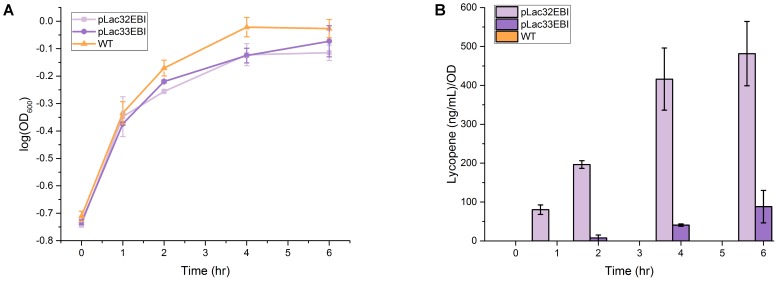
Growth curve and lycopene production of *pLac32EBI, pLac33EBI*, and wild type. **(A)** Although the two lycopene-producing strains have nearly identical growth profiles, the production of lycopene does have a minor impact on growth. **(B)** OD-normalized lycopene production is different by about an order of magnitude over most of the time course. Error bars represent standard deviation.

All three strains were grown and sampled over 6 h after inoculation and induction from a saturated overnight culture, as this time-frame and protocol are relevant to the biosensor application for which the cells are producing lycopene. The growth profiles of the strains indicate that lycopene production at levels considered in this work has a measurable but quantitatively small impact on growth under these culture conditions when compared to wild type (**Figure 1A**). This is consistent with our observations from previous work in this area. Differences in growth curves between the higher and lower-producing strains were generally insignificant. Despite the nearly identical growth profiles of the two lycopene-producing strains, their total lycopene levels differ by approximately an order of magnitude throughout most of the time course (**Figure 1B** and Supplementary Table 2), suggesting that while lycopene production can be toxic to *E. coli* growth at sufficiently high levels, the cells under study here have quite a bit of tolerance for it. Confirming this fact then allowed us to attribute subsequent differences in metabolite profiles between strains to differences in lycopene production rather than non-specific metabolic responses to toxicity.

### Culture Time and Variation in Lycopene Production Levels Affect Metabolism

To test whether this degree of variation in the production of lycopene has measurable effects on metabolism, we performed metabolomics analysis on all three strains (two lycopene producers and the wild type). Two-dimensional gas chromatography coupled to mass spectrometry (GCxGC-MS) was used to analyze the metabolic profiles of the intracellular samples collected over the 6-h time-course experiments; this technique is particularly effective for the analysis of small polar metabolites such as those in central carbon metabolism. After peak alignment, data processing, and removal of peaks that were not reproducibly measured, all 1002 remaining peaks – which included both identified and unannotated analytes – were used in subsequent analyses.

**FIGURE 2:**
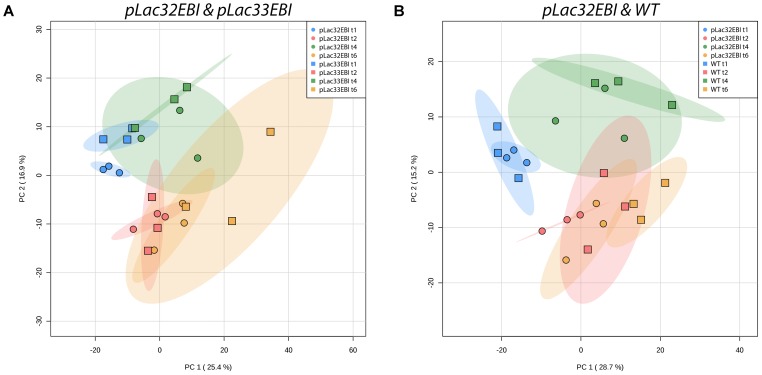
Principal Component Analysis of **(A)** *pLac32EBI* and *pLac33EBI* and **(B)** *pLac32EBI* and wild type metabolomics data. **(A)** Principal component analysis (PCA) shows little to no separation between the *pLac32EBI* and *pLac33EBI* strains across time points, indicating that variance is affected more by time than the amount of lycopene production. **(B)** There is also little to no separation between the *pLac32EBI* and wild type strains across the time course, indicating that variance is affected more by time than the production of lycopene. All colored ellipses represent 95% confidence intervals for each group.

**FIGURE 3:**
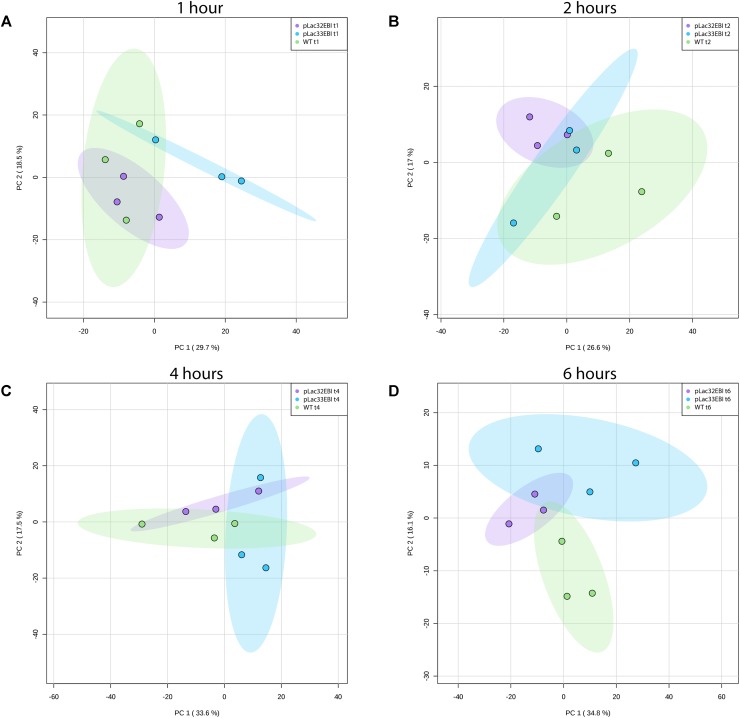
Principal component analysis of *pLac32EBI, pLac33EBI*, and wild type metabolomics data at each time-point. At **(A)** 1 h, **(B)** 2 h, and **(C)** 4 h, there is a small degree of separation between strains. At **(D)** hour 6, there is clear separation between all strains. All colored ellipses represent 95% confidence intervals for each group.

We found that the predominant effect on the metabolic profiles of each strain was the time course rather than the strain. We used Principal Component Analysis (PCA) as an unsupervised dimensional reduction approach to characterize metabolic changes across strains and time points and identify the dominant axes of variation in the dataset, along with the metabolites that most strongly contribute to those axes of variation. Directly comparing the higher and lower producers (**Figure 2A**) and the higher producer to wild type (**Figure 2B**), it is clear that the two major modes of variation, accounting for over 40% of the variability in the data, are affected more by the time in culture than by the lycopene expression levels of the strains. There is little to no separation between strains across the time course. Similar analysis of all three strains together yields the same results (Supplementary Figure 1) [The initial inoculation time point was excluded from these plots for clarity since it overlaps in PCA space for all samples (Supplementary Figure 2)]. Consistent with this, two-way Analysis of Variance on all strains for hours 1 to 6 yielded hundreds of measured analytes with a significant time effect but fewer than 100 with significant strain effects (Supplementary Table 3). While metabolic changes are dominated by time-specific changes, there are still detectable and measurable differences between the three strains metabolically. **Figure 3** shows PCA plots at individual time points, during which the three strains are easily distinguishable via their metabolic profiles at 6 h, and distinguishable to a modest extent at earlier time points. Taken together, these results indicate that metabolic changes associated with varying levels of lycopene production are, while detectable, less quantitatively significant than the changes induced as a function of time during batch growth.

We then analyzed through one-way ANOVA at each individual time point which metabolites were significantly changed across the strains. Of the individual ANOVA-significant analytes across the time points, most were unannotated, with only a handful of putatively annotated metabolites. These known metabolites did not appear to have any strong relationship to lycopene biosynthesis or other strong pathway enrichment, precluding a more detailed or mechanistic interpretation of the significantly changing metabolites. In addition, previously-reported metabolic indicators of cell stress response in *E. coli* [for example, increased production of almost all amino acids and decreases in L-alanine and L-methionine (Jozefczuk et al., 2010; Ye et al., 2012; Drazic et al., 2015)], were generally not evident in our data (with a few minor exceptions shown in Supplementary Figure 3).

### Time of Mevalonate Pathway Induction Significantly Affects Growth and Lycopene Production

While the baseline characterization of the metabolic impacts of lycopene production on the cells was important, our main goal was to study the cellular response to induction of the mevalonate pathway. The mevalonate pathway supplements the production of FPP, the last endogenous precursor to lycopene in *E. coli*. Our previous work showed that inducing the mevalonate pathway in overnight pre-culture drastically decreases both cell growth and per-cell lycopene production compared to induction at inoculation of the 6-h time course (McNerney and Styczynski, 2017a).

To study this phenomenon, we used just the higher-producing strain (*pLac32EBI*) as a model because it provided easily measurable levels of lycopene relevant for our target application but avoided significant growth toxicity. We then heterologously expressed either the lycopene production pathway, the mevalonate pathway, or both in three strains (*pLac32EBI + pBadØ*, *pLac32Ø + pBadMEV*, and *pLac32EBI + pBadMEV*, respectively). In all cases, the lycopene-producing pathway (or its null construct) was induced only at inoculation after overnight culture, while the mevalonate pathway (or its null construct) was induced either only at inoculation or starting at the overnight pre-culture.

**FIGURE 4:**
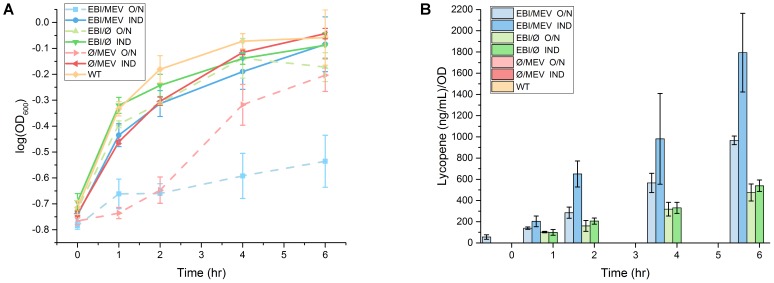
Growth profiles and lycopene production in response to overnight and inoculation induction of the mevalonate pathway. **(A)** When the mevalonate pathway is induced during overnight growth (O/N, dashed lines), mevalonate-expressing strains suffer from growth inhibition. The growth inhibition subsides at the end of the time course for the mevalonate-only strain, but persists for the strain also expressing lycopene. All strains have little to no growth inhibition when mevalonate is induced at inoculation (IND, solid lines). **(B)** OD-normalized lycopene production is greater for the mevalonate-expressing strains regardless of induction time, but overnight induction yields lower OD-normalized lycopene production than inoculation induction. Error bars represent standard deviation.

Consistent with our previous study, overnight induction of the mevalonate pathway in the lycopene-producing strain severely inhibited growth compared to induction at inoculation for the identical strain (**Figure 4A**). Even for the overnight-induced mevalonate-only strain, there was visible growth inhibition compared to induction at inoculation for the identical strain. Of note is that this growth inhibition seemed to subside between 2 and 4 h into the culture, with the cells returning to a growth rate similar to that of any other non-growth-restricted condition. Thus, overnight induction of the mevalonate pathway has some intrinsically toxic effect on cell growth that can be overcome after a sufficient recovery time, but production of lycopene prevents any such recovery and exacerbates the toxicity.

Nonetheless, this toxicity is not directly attributable to lycopene levels in the cells. Consistent with previous observations, overnight induction of the mevalonate pathway increased normalized lycopene production and decreased growth rate, while mevalonate pathway induction at the same time as lycopene pathway induction yielded even higher normalized lycopene levels and less cell toxicity (**Figure 4B** and Supplementary Table 4). Thus, potential toxicity of lycopene or its intermediates appears to be insufficient to explain the increased growth restriction due to mevalonate pathway induction.

### Time of Mevalonate Pathway Induction Significantly Affects Metabolism

**FIGURE 5:**
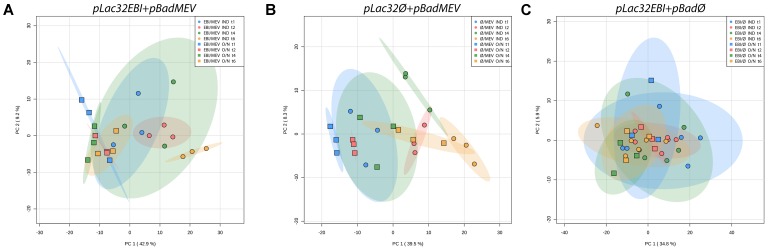
Principal component analysis of overnight- and inoculation-induced *pLac32EBI + pBadMEV, pLac32Ø + pBadMEV*, and *pLac32EBI + pBadØ* metabolomics data. **(A)** In *pLac32EBI + pBadMEV* strains, there is visible separation between induction conditions across the time course, with induction effects far outweighing temporal effects. **(B)** Separation is also seen for *pLac32Ø + pBadMEV* strains between induction conditions, though the magnitude of separation is similar to the magnitude of the temporal effects and there is less distinct separation at hour 6. **(C)** In *pLac32EBI + pBadØ* strains, there is little separation at each time point, indicating no metabolic distinction between induction conditions. “O/N” indicates overnight induction; “IND” indicates inoculation induction. All colored ellipses represent 95% confidence intervals for each group.

To characterize the underlying metabolic impact of the different heterologously expressed pathways and induction times, we performed metabolomics analysis, as described above, on the engineered strains in the two induction conditions. After peak alignment, data processing, and removal of peaks that were not reproducibly measured, all 400 remaining peaks – which included both identified and unannotated analytes – were used in subsequent analyses. PCA for samples from 1 h through 6 h clearly demonstrate the metabolic impact that the mevalonate pathway has on the cells. [Similar to the previous experiment, the initial inoculation time point was omitted from these plots for clarity since it overlaps in PCA space for all samples (Supplementary Figure 4). This similarity of initial metabolic profiles is perhaps unsurprising, as the steps leading up to this sampling, including saturation in stationary phase in depleted growth medium followed by centrifugation and resuspension during an extended time at room temperature, may have induced a common metabolic response across all of the conditions.] The *pLac32EBI + pBadMEV* strain shows essentially complete separation in the first principal component strictly by induction time (**Figure 5A**). This difference is likely not attributable just to differences in lycopene production levels, as similar separation was not seen between the higher- and lower-producing strains in **Figure 2A**. The *pLac32Ø + pBadMEV* strain also exhibits induction time-dependent group separation (**Figure 5B**), though not to the same extent; the two treatment conditions do not completely separate out in PCA space, but for any individual time point the two treatment conditions are obviously separated in the first principal component. The *pLac32EBI + pBadØ* strain, on the other hand, showed no visible separation between groups at any time point (**Figure 5C**).

Two-way ANOVA analysis results were generally consistent with what was observed in PCA. In the *pLac32EBI + pBadMEV* strain that completely separated in PCA space based on mevalonate pathway induction, 159 metabolites were significantly different across the time course based on induction time (**Table 2**). Fewer metabolites were significant for the *pLac32Ø + pBadMEV* strain (consistent with the decreased separation of induction conditions overall and separation only evident for individual time points). Since there appeared to be a major physiological change in the *pLac32Ø + pBadMEV* strain starting at 4 h, likely reverting to a state similar to inoculation induction of the mevalonate pathway, we also performed this ANOVA analysis specifically only at hours 1 and 2, as this change may have obscured group effects across the whole time course. This analysis identified 60 metabolites with significant induction condition effects. In the *pLac32EBI + pBadØ* strain, only one metabolite was significantly different, consistent with the lack of separation between these two conditions.

**Table 2:** Two-way ANOVA for overnight- and inoculation-induced *pLac32EBI + pBadMEV, pLac32Ø + pBadMEV*, and *pLac32EBI + pBadØ.*

Strain (O/N and IND)	Condition effects	Time effects	Interaction effects	Condition and time effects	Time and interaction effects	Condition, time, and interaction effects
*pLac32EBI + pBadMEV* (1–6 h)	159	6	1	9	1	2
*pLac32Ø + pBadMEV (1–6 h)*	7	81	0	71	0	0
*pLac32EBI + pBadØ (1–6 h)*	1	10	0	0	0	0
*pLac32Ø + pBadMEV (1–2 h)*	60	0	0	3	0	1

*Contributions of the individual factors are calculated using false-discovery rate (FDR) corrected p-values (<0.05).*

### Time of Mevalonate Pathway Induction Significantly Affects Individual Metabolites

We then analyzed through one-way ANOVA at each individual time point which metabolites were significantly changed across the strains. Similar to the comparison between high and low lycopene producers, no metabolites were found that were obviously related to lycopene synthesis, likely due to limitations on which metabolites are derivatizable and thus detectable via GC-MS. In addition, no metabolites involved in the mevalonate pathway were identified; these analytes should be detectable via GC-MS, so may either be below the limits of detection or not annotated due to the incompleteness of spectral libraries.

Because overnight induction of both *pLac32EBI + pBadMEV* and *pLac32Ø + pBadMEV* caused inhibited growth, for both cases we investigated whether there were changes in the levels of metabolites that have been previously reported in the literature to respond to stress conditions. As discussed above, one well-characterized *E. coli* stress response is the accumulation of amino acids (except for L-alanine and L-methionine, which typically decrease). Almost all amino acid levels in all strains and conditions were identical to those in wild type, with the exception of L-phenylalanine. In inoculation-induced *pLac32EBI + pBadMEV* strains, L-phenylalanine increased over 6 h and was significantly higher than in overnight-induced *pLac32EBI + pBadMEV* strains and wild type by the end of the experiment (Supplementary Figure 5A). In inoculation-induced *pLac32Ø + pBadMEV*, L-phenylalanine levels were significantly higher than in overnight-induced and in wild type at multiple time points, though at 6 h L-phenylalanine levels were actually significantly higher in overnight-induced *pLac32Ø + pBadMEV* than in late induced and wild type (Supplementary Figure 5B). While the generally higher levels of phenylalanine in stressed conditions may be relevant, the lack of complete consistency and the lack of similar behavior in other amino acids limits the interpretability of this observation. Another metabolite that has been previously shown to be involved in *E. coli* stress response is *N*-acetyl-L-alanine. When under heat stress, *E. coli* increases production of this metabolite (Ye et al., 2012). Interestingly, *N*-acetyl-L-alanine had a decreasing trend in both overnight- and inoculation-induced *pLac32EBI + pBadMEV* as well as in wild type, and by hour 2, concentrations in inoculation-induced *pLac32EBI + pBadMEV* were significantly lower than in overnight-induced and wild type (Supplementary Figure 5C). There were no significant differences in this metabolite in the *pLac32Ø + pBadMEV* and *pLac32EBI + pBadØ* strains. Again, this result suggests that traditional indicators of *E. coli* cellular stress are not evident in the conditions we studied.

**FIGURE 6:**
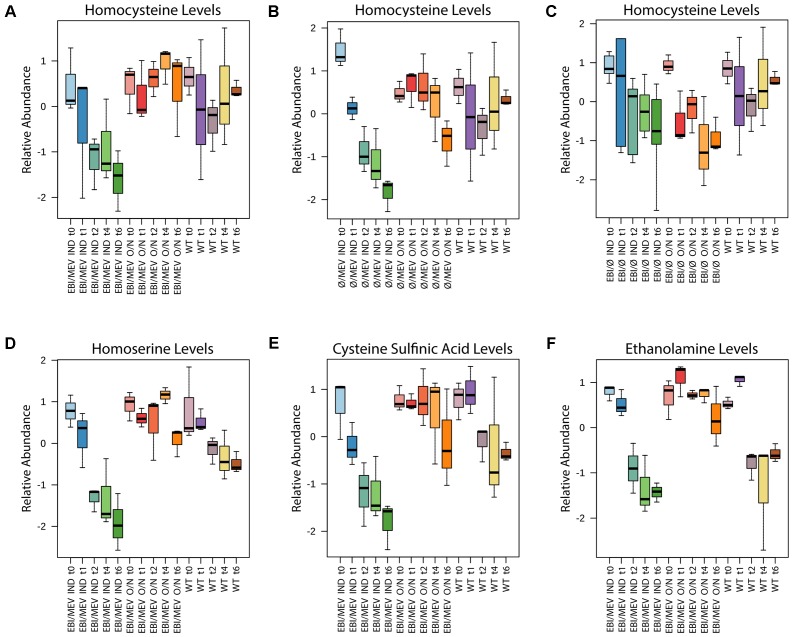
Levels of metabolites of interest plotted over time. **(A)** Homocysteine levels for overnight- and inoculation-induced *pLac32EBI + pBadMEV* strains have different trends based on induction condition. The overnight-induced strain appears to maintain higher homocysteine levels over the time course than wild type does. **(B)** Trends similar to this occur in homocysteine levels for *pLac32Ø + pBadMEV* strains at each induction condition, except in the overnight-induced strain at hour 6. **(C)** Homocysteine levels are almost identical at all time points for both overnight- and inoculation-induced *pLac32EBI + pBadØ* strains. **(D)** Homoserine levels in *pLac32EBI + pBadMEV* strains have similar trends to the ones seen in homocysteine. **(E)** Cysteine sulfinic acid and **(F)** ethanolamine trends are nearly identical to those in homoserine for *pLac32EBI + pBadMEV* strains. “O/N” indicates overnight induction; “IND” indicates inoculation induction. Box and whisker plots depict the normalized peak areas. Black lines are the medians, and boxes are the middle 50% values. Error bars represent standard deviation.

Focusing our analyses on metabolites that were most strongly affected by the time of mevalonate pathway induction, we noticed that homocysteine exhibited interesting trends. For the inoculation-induced *pLac32EBI + pBadMEV* and *pLac32Ø + pBadMEV* strains that showed no growth inhibition, there was an obvious and significant decrease in homocysteine levels over time, with levels much lower than those of wild type (**Figures 6A–C**). In contrast, the homocysteine levels in the same strains under overnight-induction, growth-inhibited conditions had similar concentrations to those in wild type at each time point (though at 6 h in the *pLac32Ø + pBadMEV* strain, when cell growth had begun to recover, homocysteine did have a downward trend compared to the earlier time points). Homocysteine in the *pLac32EBI + pBadØ* strain showed a slight downward trend regardless of induction status, consistent with the potential interplay of lycopene and mevalonate pathways’ impacts on homocysteine levels.

Homoserine, a precursor in homocysteine biosynthesis, also exhibited interesting behavior (**Figure 6D**). Similar to homocysteine, inoculation-induced *pLac32EBI + pBadMEV* had lower homoserine levels compared to overnight induction and wild type starting at 2 h after inoculation. The downward trend in homoserine was also much stronger in the inoculation-induced strain compared to wild type, with the overnight-induced strain showing no consistent trend with time. In contrast, homoserine levels had no significant changes in *pLac32EBI + pBadØ*.

Two other metabolites had profiles strikingly similar to homoserine in *pLac32EBI + pBadMEV* and wild type (**Figures 6E,F**): ethanolamine and cysteine sulfinic acid. These metabolites significantly decreased in the inoculation-induced mevalonate- and lycopene-producing strain from hours 2–6, but were comparatively constant over the entire 6 h for the same strain induced overnight. Wild type levels, on the other hand, decreased slightly by hour 2 and remained relatively constant through the rest of the time course. These metabolites did not significantly change in any other strains at either induction condition.

## Discussion

In this study, we have used GCxGC-MS metabolomics to characterize the metabolism of engineered lycopene-producing strains of *E. coli*. Previous work by our group showed that changing the RBS on lycopene biosynthesis genes had a significant effect on growth rate and lycopene production. More unexpected, though, was the fact that induction of the mevalonate pathway could have drastically different impacts on lycopene production and cell growth depending on when the pathway was induced (McNerney and Styczynski, 2017a). Yet, we had little knowledge on what metabolic differences, if any, were underlying these changes in behavior.

We surprisingly found that the metabolic impacts of lycopene production in *E. coli* cells are of much smaller magnitude than the metabolic changes inherent to simple batch growth. While we expected the lycopene-producing strains to separate clearly from the wild type strain in PCA plots, we instead found that the first principal component was dominated by temporal variation in metabolite profiles independent of lycopene production. Nonetheless, there were significant differences between the lycopene-producing strains, and analysis of individual time points, in particular the metabolite profiles at 6 h, showed that the three strains were metabolically distinct. While there were minor fluctuations in L-allothreonine, L-glutamate, and L-phenylalanine levels that have been previously associated with stress response, the behaviors were not consistent enough between strains and across potential stress-indicative metabolites to strongly suggest that the cells are under significant stress when producing lycopene at the levels studied here.

In our previous efforts, we heterologously expressed the mevalonate pathway in the same strain expressing the lycopene pathway, expecting it to improve lycopene production because it provides an alternative, non-native path to producing lycopene biosynthetic intermediates, including farnesyl pyrophosphate (FPP). While this was the case when the mevalonate pathway was induced at inoculation at the same time as induction of the lycopene pathway, overnight induction of the mevalonate pathway before inoculation caused lower cell density and smaller increases in lycopene levels, which was quite surprising. These growth profiles suggested that there may have been a toxic intermediate in the mevalonate pathway that accumulates to growth-inhibiting levels during overnight growth (Martin et al., 2003). Our metabolomics results indicate that induction time of the mevalonate pathway has a prominent impact on metabolism, especially when paired with lycopene biosynthesis.

Overnight mevalonate induction clearly has a growth and metabolic impact on cells regardless of whether the cells also express lycopene. When cells do express lycopene, the difference between overnight and inoculation induction of the mevalonate pathway is the most significant source of variation in the data, overwhelming the temporal variability associated with batch growth time-course measurements (**Figure 5A**). This is in itself noteworthy, as changes in metabolism associated just with time-course batch growth are actually greater in magnitude than the differences between strains that have significant vs. zero lycopene production (**Figure 2B**). It is quite surprising that the impact of whether the mevalonate pathway had been induced in a saturated overnight inoculation culture would be greater than the impact of whether or not cells express a heterologous pathway known to exert significant stress on cell growth and resources.

When cells do not express lycopene, the differences between inoculation and overnight induction of the mevalonate pathway are still striking and easily detectable, though on the same order of magnitude in principal component space as the temporal variations. Moreover, by the end of our sampling period (6 h), the metabolic profiles of the overnight- and inoculation-induced cultures begin to converge again (**Figure 5B**), suggesting that the cells are metabolically recovering from the lingering toxicity associated with overnight mevalonate induction. Importantly, this is consistent with the growth kinetics we observed, where cell growth increased rapidly in the overnight-induced culture starting at 4 h, with nearly-recovered cell density at 6 h.

Thus, there is something about lycopene biosynthesis (which, at the levels studied in this paper were minimally toxic and caused only small measurable differences in metabolic profiles) that particularly exacerbates the lingering toxicity associated with overnight mevalonate induction. Without lycopene production, the cells can eventually recover both metabolically and in terms of growth kinetics, but lycopene production is sufficiently antagonistic with the mevalonate pathway to push the cells to an exceedingly large metabolic deviation and prevent any growth recovery.

**FIGURE 7:**
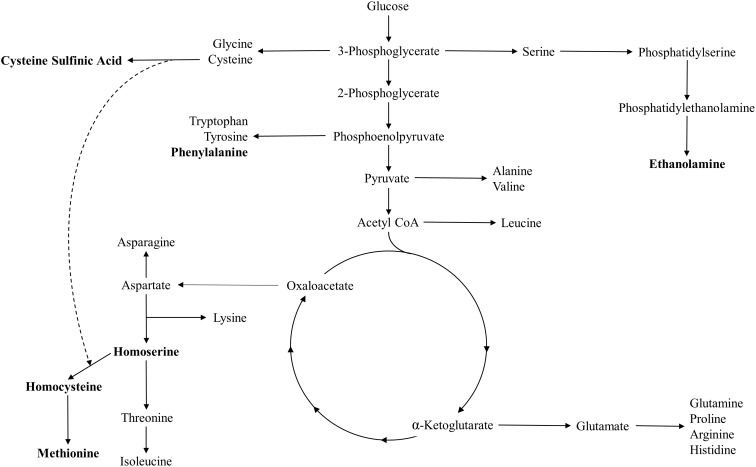
An overview of metabolic pathways relevant to this work; metabolites of interest specifically noted in this paper are bolded.

This analysis also identified two notable metabolites, homocysteine and homoserine, that could play a role in the growth inhibition seen in overnight induction of the mevalonate pathway. These metabolites are precursors to methionine biosynthesis, and are depicted in a metabolic pathway overview diagram along with other metabolites discussed in this paper in **Figure 7**. Homocysteine has previously been found to inhibit *E. coli* growth when in abundance intracellularly and extracellularly (Roe et al., 2002; Tuite et al., 2005). The drastic decrease seen in homocysteine in inoculation-induced *pLac32EBI + pBadMEV* and *pLac32Ø + pBadMEV* could possibly suggest that these strains, when induced during inoculation, are more equipped to utilize or deplete this metabolite, although the mechanism for such a difference remains unclear. Additionally, the stable trend in homocysteine levels from hours 0 to 4 in the two overnight-induced strains could indicate that early induction of the mevalonate pathway may cause the strains to have a decreased ability to handle homocysteine accumulation, which affects their growth rate. Moreover, the fact that homocysteine levels drop precipitously in the *pLac32Ø + pBadMEV* strain at 6 h, just as the cells have phenotypically recovered to high growth rates, supports the potential importance of this metabolite in mevalonate-induced toxicity. We note, however, that the wild type homocysteine levels are only slightly lower than the overnight-induced strains, complicating the direct interpretation of the importance of homocysteine levels.

The same study that reported intracellular homocysteine toxicity also noted that in their attempts to relieve this toxicity, the addition of extracellular homoserine caused intensified inhibition (Roe et al., 2002). Our results could then alternatively suggest that the inoculation-induced *pLac32EBI + pBadMEV* strains can better handle homoserine accumulation compared to the overnight-induced counterpart strain. Homoserine’s predominantly constant and high concentrations that are only visible in the overnight-induced *pLac32EBI + pBadMEV* strain may contribute to the strain’s intensified inhibition even compared to the growth profile of early induced *pLac32Ø + pBadMEV*, which does not have significant variations in homoserine compared to its counterpart strain and wild type.

To test whether homocysteine was contributing to the cytotoxicity seen in overnight-induced *pLac32EBI + pBadMEV* and *pLac32Ø + pBadMEV* strains, we conducted a medium supplementation experiment. Previous work has shown that methionine supplementation can reduce homocysteine-associated toxicity (Roe et al., 2002). We thus supplemented the medium with 2 mM methionine at inoculation. With addition of methionine, overnight-induced *pLac32EBI + pBadMEV* displayed growth improvements within 2 h compared to the same strain under the same induction conditions without methionine (Supplementary Figure 6A). Interestingly, methionine supplementation did not appear to provide significant growth benefits to the *pLac32Ø + pBadMEV* strain (Supplementary Figure 6B). Although this indicates that homocysteine accumulation is not the sole contributor to the observed toxicity, these results still imply homocysteine contributes to the growth inhibition and likely to the antagonistic effect between the mevalonate and lycopene pathways, and is thus likely useful to pursue as a target for further strain engineering.

Taken together, these observations suggest the role of a diverse set of metabolites and pathways in the different growth inhibition and metabolic phenotypes we observed. We note, however, that our analysis did not identify as significant many metabolites known to have a direct role in lycopene or mevalonate synthesis. This is likely due in part to limitations in our choice of analytical instrumentation, the GC-MS, as not many such metabolites were even annotated in our dataset. The metabolites in lycopene biosynthesis pathways have few, if any, good leaving groups for derivatization by MSTFA, which would leave them not particularly volatilizable even after derivatization and thus not easily detected by our instrument. Mevalonate pathway metabolites (such as HMG-CoA, IPP, and mevalonate) would be expected to be derivatizable, so if these metabolites are present above the detection limits of our instrument, they may appear as unknown analytes: the metabolite databases used for spectral matching are not necessarily complete for these specific pathways, and we set our identification matching thresholds conservatively to prevent incorrect metabolite annotation.

We also note some inherent limitations in our data. As noted in the Methods section, there were detectable batch effects in the mevalonate induction experiments. We used batch correction software to remove most of the systematic effects in our data, but the batch effects induced an increase in variability for biological replicates which contributed to a decreased number of analytes with significant time effects in ANOVA analysis and that dampened the visibility of the temporal variation in metabolite profiles. These batch effects are also the likely cause for the decrease in the number of properly aligned and tracked analytes in the induction experiments compared to the initial experiments looking only at different lycopene producers. Nonetheless, trends in individual analytes are consistent across mass spectral acquisition days and across experimental replicate days, supporting the validity of our results.

In addition, our data do not capture quantitative concentrations of metabolites nor identify the mechanisms driving these metabolic divergences. Despite these limitations, our ability to identify fold-changes among metabolites and pathways led us to identify key trends occurring due to heterologous pathway induction. Isotope labeling-based absolute quantification of metabolite levels and systems-scale measurement of gene expression are promising next steps to further elucidate the underlying mechanisms of the trends we identified here. Isotope labeling-based quantification could also help to identify the amount of metabolite leakage during sample quenching to validate that our modified quench protocol provides leakage comparable to the original protocol. Metabolite leakage has not explicitly been tested for and validated in this work; however, since our modified protocol yields a quenched sample with the same ethanol and salt concentrations as the original protocol (just more quickly brought to a cold temperature), we expect the metabolite leakage to be similar. Nonetheless, validation of this hypothesis would help reinforce the broader utility of the modified protocol.

An important caveat in interpreting our results is in our objective, and thus our approach, for strain design and culture. Our primary goal was to engineer strains that produce enough lycopene to visibly turn the cells red in the shortest amount of time possible for a diagnostic readout, not to produce the greatest amount of lycopene in an indefinite timeframe (which often entails significant culture time in stationary phase for non-growth-associated production of lycopene). This is the reason our maximum lycopene production rate of 291.4 [(ng/mL)/OD]/hr from the inoculation-induced *pLac32EBI + pBadMEV* strain is orders of magnitude less than values seen in the literature, which approach 0.030 mg/mL/hr (Alper et al., 2006; Zhu et al., 2015; Xu et al., 2018) over 24 h of culture. We also note that our experiments were done in the nutrient-rich LB medium. Rich media are commonly used for culturing lycopene-producing *E. coli* in order to achieve optimal production rates (Alper et al., 2006; Yoon et al., 2006; Xu et al., 2018), but rich media may affect nutrient uptake and metabolism, which we had sought to study. However, rich medium is a reasonable model for the final assay mixture in our application, which will contain 25–100% human serum – itself a complex and rich mixture of metabolites. As a result, while these design choices may slightly hinder the generalizability of our results and mechanistic interpretations, they are the most relevant for the target application and the system we will ultimately look to optimize.

## Conclusion

We have presented the first profiling of the metabolic differences caused by induction time variation of the mevalonate pathway, explored its potential relationship to lycopene production, identified a possible connection to homocysteine- and homoserine-caused growth inhibition, and validated the involvement of homocysteine-induced toxicity in our system. We also improved the existing metabolomics sampling protocols for *E. coli* cultures to minimize the amount of time cells spent at above-freezing temperatures that could lead to changes in metabolite profiles. While the underlying mechanisms of the negative effects caused by overnight induction of the mevalonate pathway are still not immediately evident, our efforts have moved us toward a better understanding of the metabolic impacts of this phenomenon and generated hypotheses that could drive future studies. In particular, our work demonstrates the power of metabolomics in helping to provide the understanding needed to drive pathway and strain engineering, and the potential utility of unraveling the mechanisms of homocysteine- and homoserine-related toxicity to allow improved carotenoid biosynthesis.

## Author Contributions

AM carried out the metabolic experiments, analyzed the samples with GCxGC-TOFMS, performed the metabolomic data analysis, and drafted the manuscript. MM constructed the strains for the experiment, performed lycopene extraction, and performed the HPLC analysis. MS and MM conceived of the study, participated in its design and coordination, and helped to draft the manuscript. All authors read and approved the final manuscript.

## Conflict of Interest Statement

MM and MS have filed a PCT patent application (No.: PCT/US2016/037542) covering pigment-based whole-cell zinc biosensors and associated applications. MS has formed a company to explore commercialization of technology in the above application. The other author declares that the research was conducted in the absence of any commercial or financial relationships that could be construed as a potential conflict of interest.

## Footnotes

1 http://metaopen.sourceforge.net/metpp.html

2 http://metaboanalyst.ca/
