# Supplementary material for: Metabolomics Analysis of the Toxic Effects of the Production of Lycopene and Its Precursors
Source: Front Microbiol. 2018 May 3;9:760. doi: 10.3389/fmicb.2018.00760 (PMC5944366; doi:10.3389/fmicb.2018.00760)
Supplement: Supplementary file 1 [file Data_Sheet_1.docx]

Supplementary Material

Metabolomics Analysis of the Toxic Effects of the Production of Lycopene and its Precursors

April M. Miguez, Monica P. McNerney, Mark P. Styczynski

## GCxGC-MS Methods

### Auto-sampler Method

An Agilent 7683 auto sampler was used. Prior to sample injection, three pre-washes were performed with pyridine. Sample was pumped 4 times for thorough mixing and injected using a syringe size of 10 µL with 1 µL of injection volume. Three post-washes of the needle were performed after injection using pyridine.

### GCxGC Method

An Agilent 7890 gas chromatograph adapted to GCxGC analysis was used. Helium was used as the carrier gas with a corrected constant flow rate of 1.00 mL/min. The inlet septum purge flow was maintained at 3 mL/min. The inlet was functioning in splitless mode with a purge flow of 100 mL/min delayed to start 30 seconds after injection, giving a total flow of 101 mL/min. Runs were performed in a gas saver mode with a flow of 20 mL/min set to start a minute after injection. Front inlet temperature was set at 250°C for the entire run.

The primary oven temperature was held at 70°C for 1 min and the temperature was ramped at 10°C/min until 315°C and held for 2 minutes. The secondary oven temperature and the modulator temperature offsets were 5°C and 15°C above the main oven respectively. One minute equilibration time was set for the ovens. The modulation program is listed in Supplementary Table 1. The transfer line temperature was maintained at 320°C for the entire run.

Supplementary Table 1. Modulation Timing

| # | Start (s) | End (s) | Modulation period (s) | Hot pulse time (s) | Cool time between stages (s) |
| --- | --- | --- | --- | --- | --- |
| 1 | Start | 392 | 6.00 | 1.00 | 2.00 |
| 2 | 392 | End of Run | 6.00 | 1.50 | 1.50 |

**MS Method**

A Leco Pegasus 4D time of flight mass spectrometer (TOF-MS) with electron impact ionization was used for mass analysis. Filaments were turned off for the initial 230 seconds to delay mass acquisition until after the solvent peak. The mass scanning range was from 50 to 500 u with an acquisition rate of 200 spectra per second. The detector voltage was set at 100 V above the optimized voltage with an electron energy of -70 V. Manual mass defect mode was used with the mass defect 0 mu/ 100 u. Ion source temperature was required to reach 220°C before starting mass acquisition.

**Supplementary Table 2.** OD-normalized lycopene production for the high and low lycopene producing strains *pLac32EBI* and *pLac33EBI* at each time point

| **Strain** | **Lycopene (ng/mL)/OD** | | | | | **Lycopene Production Rate ((ng/mL)/OD)/hr** |
| --- | --- | --- | --- | --- | --- | --- |
|  | **0 hr** | **1 hr** | **2 hr** | **4 hr** | **6 hr** |  |
| *pLac32EBI* | 0 | 80.3 ± 12.2 | 196.3 ± 9.8 | 416.5 ± 79.9 | 481.5 ± 82.6 | 85.1 |
| *pLac33EBI* | 0 | 0 | 7.6 ± 7.6 | 40.6 ± 3.0 | 88.2 ± 41.7 | 15.2 |

**Supplementary Table 3.** Two-way ANOVA for *pLac32EBI* and *pLac33EBI* as well as *pLac32EBI* and wild type from 1-6 hours

|  | **Group Effects** | **Time Effects** | **Group & Time Effects** | **Interaction Effects** |
| --- | --- | --- | --- | --- |
| *pLac32EBI* and *pLac33EBI* | 84 | 290 | 7 | 0 |
| *pLac32EBI* and wild type | 50 | 360 | 29 | 0 |

**Supplementary Table 4.** OD-normalized lycopene production and rate for lycopene producing strains *pLac32EBI+pBadMEV* and *pLac32EBI+pBadØ* at each time point

| **Strain** | **Lycopene (ng/mL)/OD** | | | | | **Lycopene Production Rate ((ng/mL)/OD)/hr** |
| --- | --- | --- | --- | --- | --- | --- |
|  | **0 hr** | **1 hr** | **2 hr** | **4 hr** | **6 hr** |  |
| *EBI/MEV* (O/N) | 55.7 ± 21.5 | 138.8 ± 10.9 | 284.9 ± 52.9 | 566.0 ± 90.6 | 967.0 ± 40.3 | 152.7 |
| *EBI/MEV* (IND) | 0 | 203.2 ± 50.2 | 650.0 ± 122.3 | 981.4 ± 427.9 | 1794.4 ± 370.9 | 291.4 |
| *EBI/Ø (O/N)* | 0 | 101.9 ± 6.9 | 159.8 ± 51.2 | 318.0 ± 64.6 | 475.6 ± 79.8 | 77.7 |
| *EBI/Ø (IND)* | 0 | 99.3 ± 27.0 | 205.7 ± 29.5 | 330.8 ± 52.0 | 539.0 ± 54.2 | 86.8 |


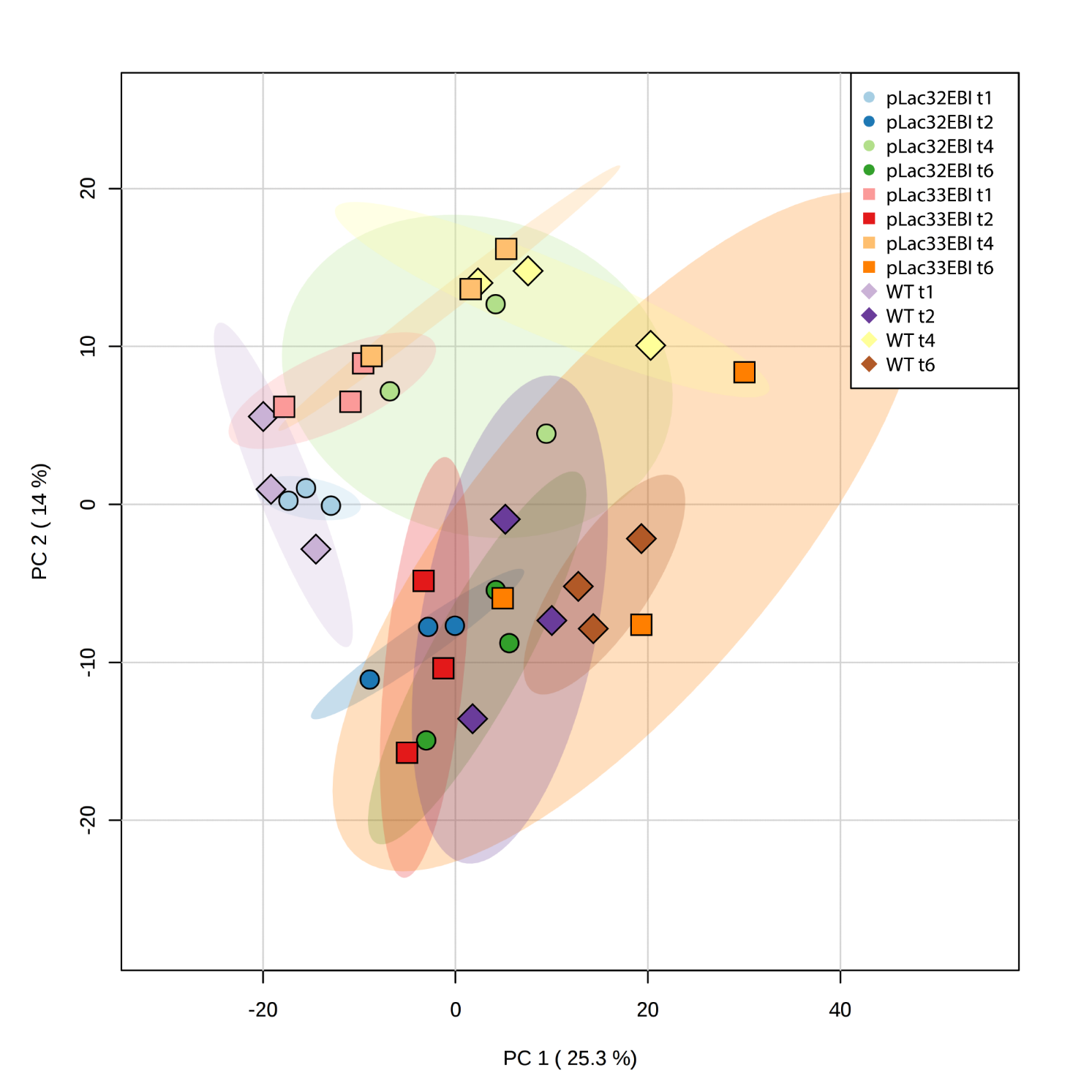


**Supplementary Figure 1.** PCA of *pLac32EBI, pLac33EBI,* and wild-type metabolomics data. PCA of all three strains indicate that there is little to no separation between strains across the time course. All colored ellipses represented 95% confidence intervals for each group.

**
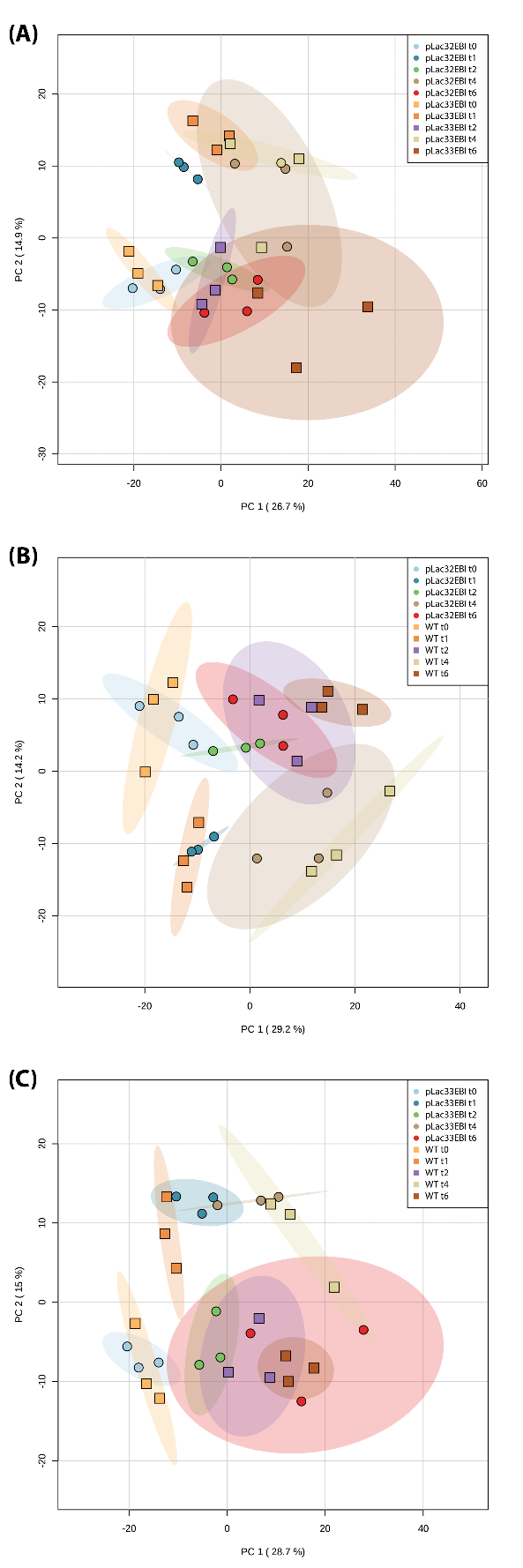
**

**Supplementary Figure 2.** PCA of (A) *pLac32EBI* and *pLac33EBI,* (B) *pLac32EBI* and wild-type, and (C) *pLac33EBI* and wild type metabolomics data at all time points. In all analyses, strain groups at the initial inoculation time point do not separate from one another. Based on this, the initial inoculation time point was excluded in subsequent PCA analyses and from figures in the main text. All colored ellipses represent 95% confidence intervals for each group.


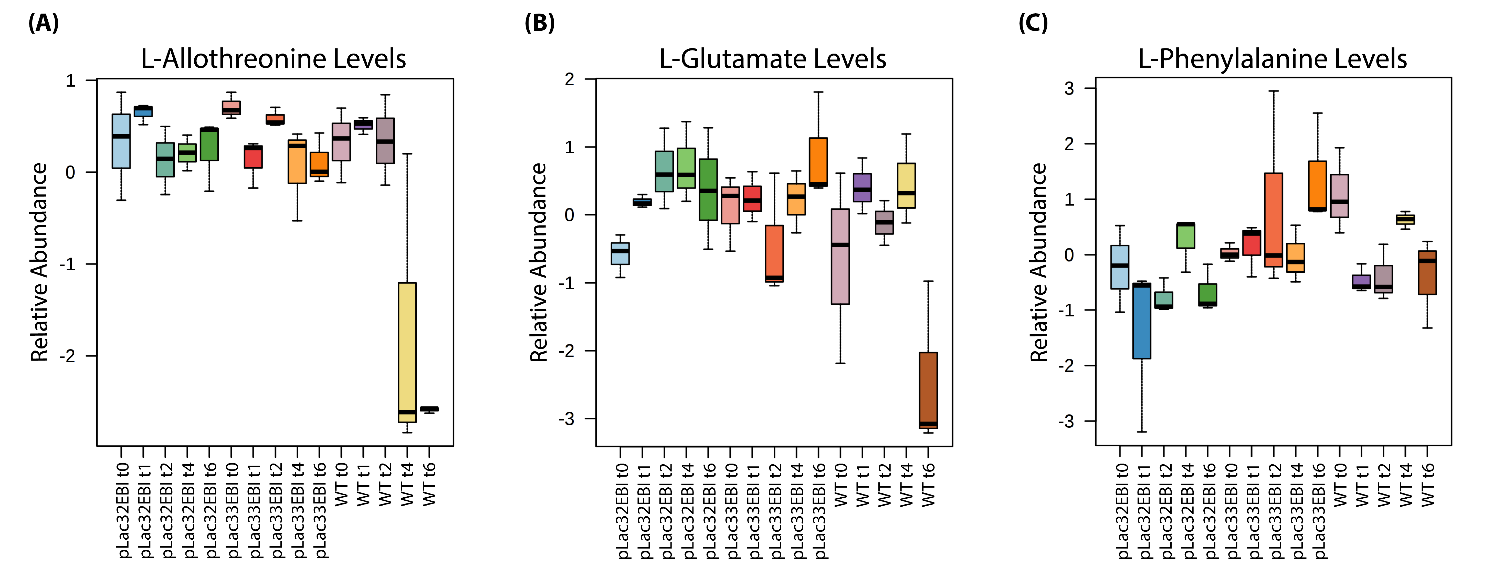


**Supplementary Figure 3.** Profiles of some amino acids of potential interest. (A) L-allothreonine decreased in wild type but not in the lycopene-producing strains. (B) This trend was also seen for L-glutamate levels. (C) At hour 6, *pLac33EBI* had higher L-phenylalanine levels than the other two strains. Box and whisker plots depict the normalized peak areas. Black lines are the medians, and boxes are the middle 50% values. Error bars represent standard deviation.

**
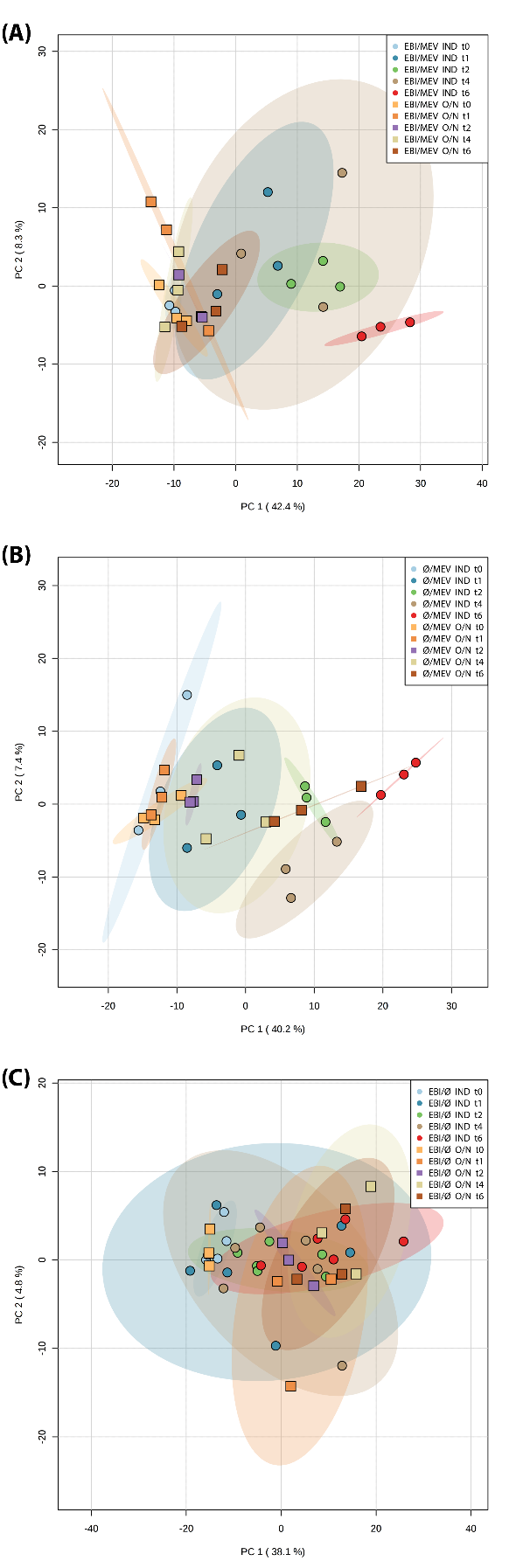
**

**Supplementary Figure 4.** PCA of overnight- and inoculation-induced (A) *pLac32EBI+pBadMEV,* (B) *pLac32Ø+pBadMEV,* and (C) *pLac32EBI+pBadØ* metabolomics data. The initial inoculation time point overlaps in PCA space for all samples, so was excluded from plots in the main text for clarity. “O/N” indicates overnight induction; “IND” indicates inoculation induction. All colored ellipses represent 95% confidence intervals for each group.


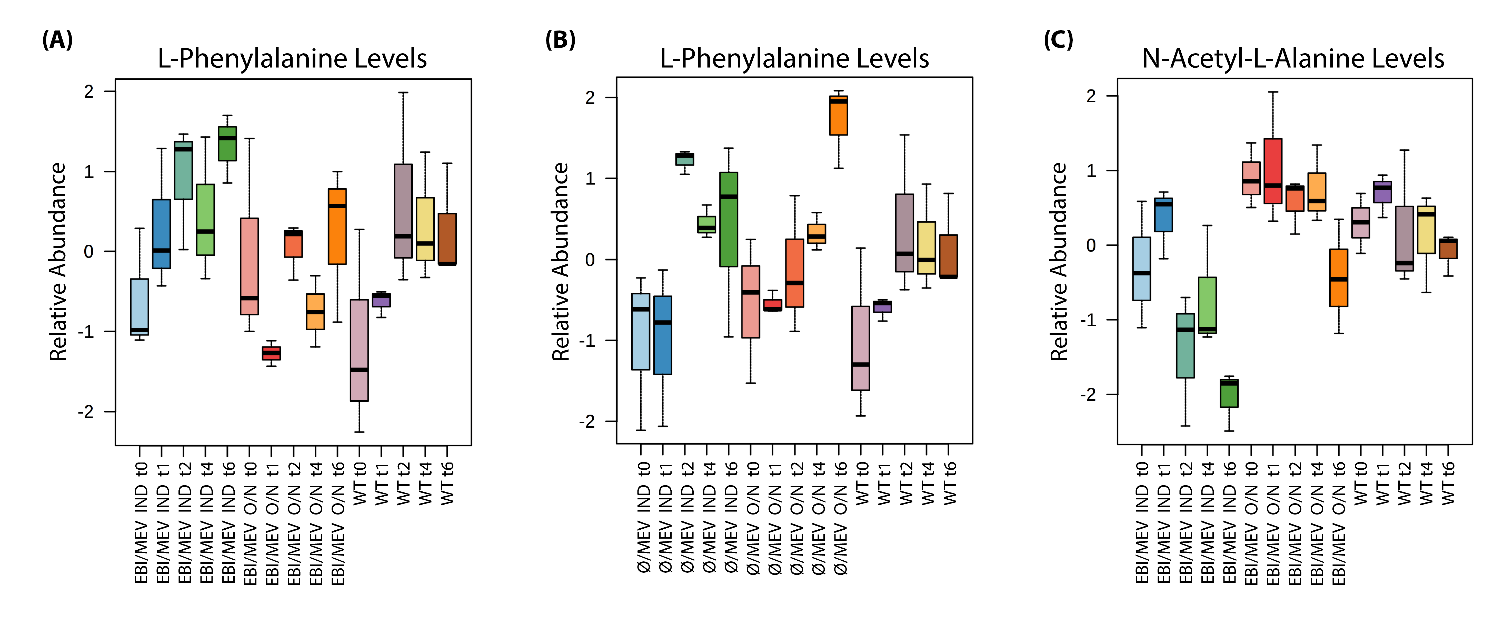


**Supplementary Figure 5.** Profiles of stress-associated metabolites. (A) L-phenylalanine increased over 6 hours and was significantly higher in inoculation-induced *pLac32EBI+pBadMEV* strains than in overnight-induced *pLac32EBI+pBadMEV* strains and wild type by the end of the experiment. (B) L-phenylalanine levels were significantly higher in inoculation-induced *pLac32Ø+pBadMEV* than in overnight-induced or in wild type at multiple time points. However, at 6 hours L-phenylalanine levels were significantly higher in overnight-induced *pLac32Ø+pBadMEV* than in late-induced and wild type. (C) N-acetyl-L-alanine had a decreasing trend in both overnight- and inoculation-induced *pLac32EBI+pBadMEV* as well as in wild type, but to substantially different degrees. By hour 2, concentrations in inoculation-induced *pLac32EBI+pBadMEV* were significantly lower than in overnight-induced and wild type. “O/N” indicates overnight induction; “IND” indicates inoculation induction. Box and whisker plots depict the normalized peak areas. Black lines are the medians, and boxes are the middle 50% values. Error bars represent standard deviation.


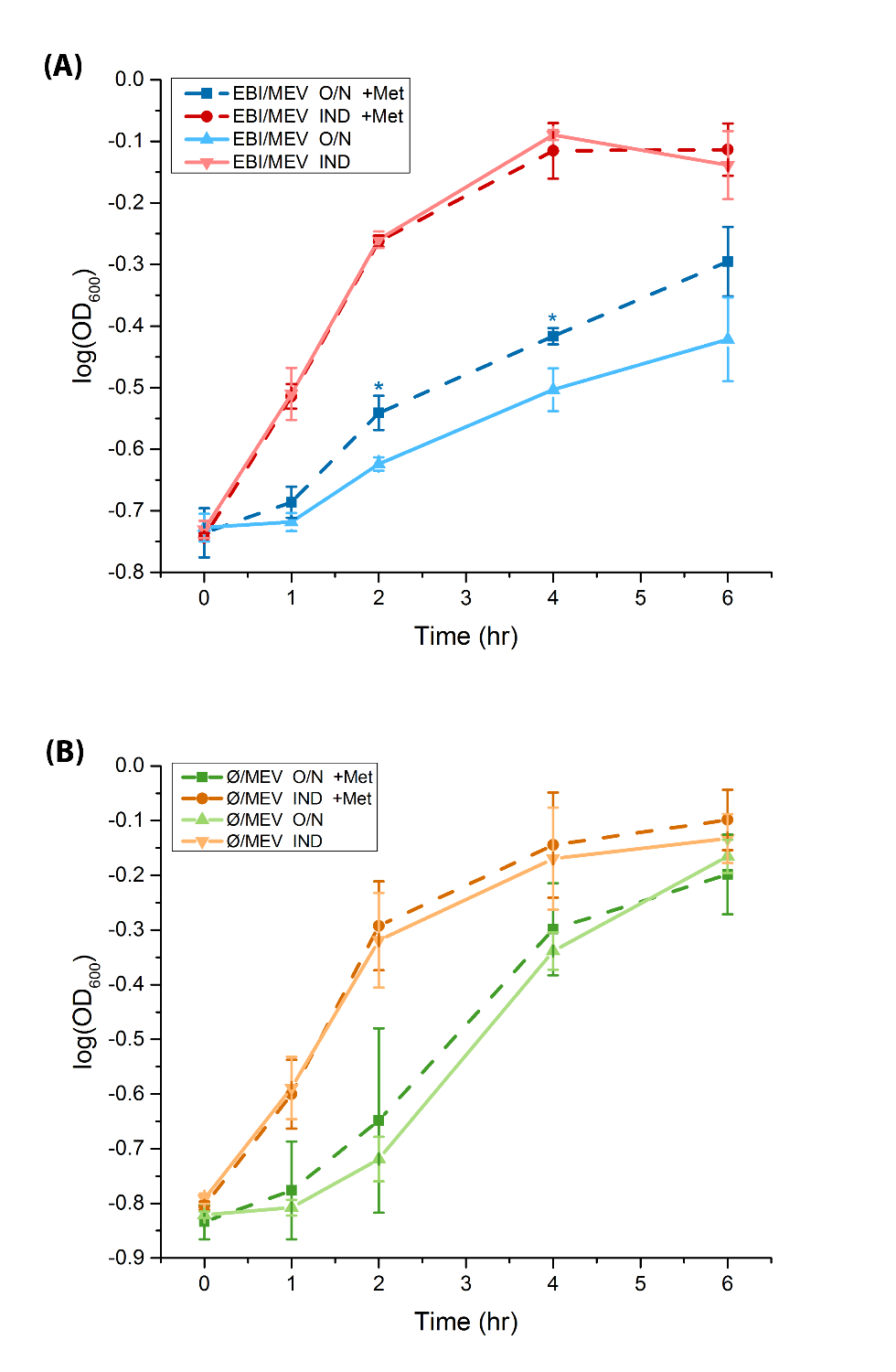


**Supplementary Figure 6.** Growth profiles in response to 2mM methionine supplementation. (A) When the medium is supplemented with 2 mM methionine, the growth inhibition of the *pLac32EBI+pBadMEV* strain induced with arabinose overnight (blue dashed line) is partially alleviated compared to the same strain under the same induction conditions without methionine (blue solid line). Methionine addition has no significant effect on the growth of the *pLac32EBI+pBadMEV* strain induced at inoculation (red dashed and solid lines). (B) Methionine supplementation for overnight-induced (green dashed line) and inoculation-induced (orange dashed line) *pLac32Ø+pBadMEV* strains did not significantly improve growth compared to the same strain under the same induction conditions without methionine (green and orange solid lines), though there does seem to be an insignificant trend towards alleviation at earlier time points which is counteracted by the cells’ normal recovery at later time points. “O/N” indicates overnight induction; “IND” indicates inoculation induction; “+Met” indicates supplementation of 2mM methionine. Error bars represent standard deviation. Asterisks indicate statistically significant differences with a p-value < 0.05.
